# Transcriptomic profiling of human endothelial cells infected with venezuelan equine encephalitis virus reveals NRF2 driven host reprogramming mediated by omaveloxolone treatment

**DOI:** 10.3389/fgene.2025.1722527

**Published:** 2025-12-18

**Authors:** Mostafa Rezapour, Lorreta A. Opoku, Stephanie V. Trefry, Abbas Alili, Maame Konadu, Maria Galarza Dionisio, Metin Nafi Gurcan, Aarthi Narayanan

**Affiliations:** 1 Wake Forest Institute for Regenerative Medicine, Wake Forest University School of Medicine, Winston-Salem, NC, United States; 2 Department of Biology, George Mason University, Fairfax, VA, United States; 3 Center for Artificial Intelligence Research, Wake Forest University School of Medicine, Winston-Salem, NC, United States

**Keywords:** venezuelan equine encephalitis virus (VEEV), omaveloxolone (OMA), Nrf2 signaling, transcriptomics, host-directed therapy, generalized linear model with quasi-likelihood and magnitude-altitude scoring (GLMQL-MAS)

## Abstract

**Introduction:**

Venezuelan Equine Encephalitis virus (VEEV) is a mosquito-borne alphavirus that causes neurotropic disease with significant morbidity and mortality, especially in children. While interferon-stimulated genes (ISGs) are central to host defense, therapeutic modulation of host responses remains underexplored. Omaveloxolone (OMA), an FDA-approved NRF2 activator, has been proposed as a candidate for host-directed antiviral therapy.

**Methods:**

We investigated transcriptomic responses of human umbilical vein endothelial cells (HUVECs) infected with VEEV TC-83 in the presence or absence of OMA at 24 hours post-infection using RNA-Seq. Differential expression analysis was performed with Generalized Linear Model with Quasi-Likelihood and Magnitude-Altitude Scoring (GLMQL-MAS), followed by Cross-MAS to distinguish shared and condition-specific programs.

**Results:**

Untreated VEEV infection induced a canonical ISG signature including *IFIT1-3, OASL, RSAD2*, and *MX1*, together with cytokine and chemokine signaling (*IL6, CXCL10, CXCL11*), consistent with a strong proinflammatory and antiviral state. In contrast, OMA treatment elicited a broader shift, with 729 upregulated and 1,264 downregulated genes. Key OMA-induced genes (*HMOX1, NQO1, GCLM, TXNRD1, SLC7A11*) mapped to NRF2-dependent antioxidant, ferroptosis resistance, and detoxification pathways, accompanied by widespread repression of histone cluster genes. Cross-MAS revealed 695 OMA-unique upregulated genes, 86 untreated-unique genes, and 34 shared genes forming a compact interferon-centered antiviral backbone. Network analyses highlighted NRF2-driven antioxidant modules under OMA and cytokine-chemokine modules under untreated infection.

**Discussion:**

These findings demonstrate that OMA redirects host transcription from an interferon-centric, inflammatory response toward an NRF2-driven cytoprotective program while preserving core antiviral mechanisms, which supports NRF2 activation as a therapeutic strategy against VEEV.

## 1 Introduction

Venezuelan Equine Encephalitis virus (VEEV) is an alphavirus transmitted by the bite of mosquitoes that causes neurotropic and encephalitis diseases and can lead to death (Jo et al., 2025; Sreepangi et al., 2024; Vittor et al., 2016). Overall case-fatality is under 1%, but in children under 15, about 5% develop neurological disorders, of which about 10% are fatal and may lead to epilepsy, paralysis, or other lasting impairments (Jo et al., 2025; Guzmán-Terán et al., 2020). Host factors often play an important role in virus infection, which makes the identification and characterization of host factors valuable for insight into viral pathogenesis and potential broadly effective strategies to develop antiviral therapeutics (Xie et al., 2024). Alphavirus replication depends on host factors such as *G3BP1/2*, *FXR1/2*, *FMR*, *SEC61A*, *VCP*, and *FHL1*, while ISGs including *IFIT1*, *IFIT3*, and *ISG20* act as restriction factors (Boghdeh et al., 2023; Kim et al., 2016; Meertens et al., 2019; Panda et al., 2013; Reynaud et al., 2015).

Omaveloxolone (OMA) is an NRF2 modulator which is FDA-approved. OMA reduces viral load both extracellularly and intracellularly by modulating antiviral and anti-inflammatory responses through the modulation of ubiquitination of the NRF2 signaling pathway (Bhadoria et al., 2021). Although NRF2 biology and the concept of host directed therapy are increasingly appreciated, a systems level definition of how VEEV reshapes the transcriptome in human endothelial cells and how OMA modifies those programs is still underexplored. Endothelial cells are central to vascular integrity and trafficking of immune cells, which places them at the interface between viral pathogenesis and host defense.

We previously characterized the antiviral activity of OMA and related ubiquitin–proteasome pathway modulators against New World alphaviruses in conventional cell culture models and in a three-dimensional human neurovascular unit (NVU) organ-on-a-chip system (Boghdeh et al., 2023; Boghdeh et al., 2022). Those studies established that OMA decreases infectious titers and viral RNA in a dose-dependent manner across multiple CNS-relevant cell types while maintaining non-cytotoxic concentration ranges defined by viability assays (Boghdeh et al., 2023), and that OMA lowers viral burden, limits cytokine production, and preserves barrier properties in an NVU platform that recapitulates the multicellular blood–brain barrier microenvironment (Boghdeh et al., 2022). Our group also demonstrated that VEEV infection alters mitochondrial function, redox balance, and inflammatory signaling in CNS cells, which provides additional context for understanding host-directed interventions in VEEV infection (Keck et al., 2018; Keck et al., 2017). Together, these studies provide a foundation for defining how OMA reprograms host responses at the transcriptomic level.

In the current study, we address this gap by profiling the transcriptomic response of primary human umbilical vein endothelial cells infected with VEEV TC-83 in the presence or absence of OMA at 24 h post-infection (hpi) using RNA-Seq. We apply Generalized Linear Model with Quasi-Likelihood and Magnitude-Altitude Scoring (GLMQL-MAS) framework (Rezapour et al., 2024a; Rezapour et al., 2024b; Rezapour et al., 2024c), which is an EdgeR based model, to identify differentially expressed genes. We then integrate a cross comparison ranking method, Cross-MAS (Rezapour et al., 2024d), which distinguishes genes that are common to both conditions from those that are unique to OMA treatment or to untreated infection, and we contextualize these sets with functional enrichment analyses.

The contributions of this study are fourfold. First, we provide a comprehensive atlas of VEEV-induced transcriptional changes in human endothelial cells at an early time point, which defines the core antiviral backbone that is shared across conditions. Second, we delineate the OMA-specific signature that concentrates on redox control, detoxification, and ferroptosis restraint, which clarifies how NRF2 activation rebalances host responses during infection. Third, we map the untreated infection response enriched for cytokine and chemokine signaling and endothelial activation, which explains inflammatory features that accompany alphavirus infection. Fourth, we introduce an analysis framework that couples quasi likelihood modeling with cross condition ranking and pathway mapping, which can be reused to evaluate other host directed interventions. Together, these elements establish a clear rationale for NRF2-directed therapy in VEEV infection, which provides mechanistic anchors that motivate future validation and therapeutic testing.

## 2 Materials

### 2.1 Cell culture

Human umbilical vein endothelial cells (HUVEC) (Lonza, Cat# C2517A) were cultured and seeded at 300,000 cells per well in 6-well plates (Gene Clone cell culture, Cat# 25-105) and maintained in EBM-2 Basal Medium supplemented with EGM-2 Microvascular Endothelial Cell Growth Medium SingleQuots, as required for growth (Lonza, Cat# CC-3156). Vero African Green Monkey kidney epithelial cells (ATCC, Cat# CCL-81) were cultured and seeded in 12-well plates (Greiner bio-one CellStar Cat# 665180) at 1.6 × 10^5^ per well for 24 h and maintained in cell culture medium Dulbecco’s Modified Eagle Medium (DMEM) with L-glutamine (ATCC, Cat# 112-014–101) supplemented with 1% Penicillin streptomycin (Corning, Cat# 30-002-CI) and 10% heat-inactivated Fetal Bovine Serum (Gibco, Cat#A52568-01). Cells were continually maintained at 37 °C and 5% CO_2_ culture conditions throughout the experiments.

### 2.2 Virus and infection

Venezuelan Equine Encephalitis Virus (VEEV) TC-83 strain was generated using a genomic clone that was kindly provided by Dr. Frolov (University of Alabama at Birmingham). Cultured cells were pre-treated with OMA-containing media at 0.5 μM for 1 h, removed, and infected at a multiplicity of infection (MOI) of 0.1 with VEEV TC-83. The inoculum was removed after 1 h of infection and replaced with fresh media containing OMA at 0.5 μM. Culture supernatants were collected at 24 h post-infection (hpi) and analyzed by plaque assay.

### 2.3 Virus titration

Vero cells were plated in 12-well plates to reach ∼70–80% confluence overnight at 37 °C, 5% CO_2_. Supernatant samples were diluted in DMEM from 10^−1^ to 10^−8^, and infection was carried out for each dilution as described above. At 1 h post-infection, 1 mL of a 1:1 solution of 0.6% agarose in distilled water with 2x Eagle’s Minimal Essential Medium (ATCC, Cat# 115-073–101), supplemented with 2% Penicillin streptomycin, 5% heat-inactivated Fetal Bovine Serum, 1% Sodium pyruvate (Corning, Cat# 25-00-CI), 1% Minimum Essential Media-Non-essential amino acids (100X) (ATCC, Cat# 116-078–721) and 1% L-glutamine (Gibco, Cat# 25030-081) was added to each well. Plates were allowed to solidify at room temperature and subsequently transferred to a humidified incubator at 37 °C, 5% CO_2_ and incubated for 48 h. At the end of the incubation time, plates were fixed with 10% formaldehyde (VWR Cat# 77507-012) overnight. After fixation, the agar plugs were discarded, and plates were stained with 1% crystal violet (Thermo, Cat# 405835000) in 20% ethanol (VWR, Cat# 77507-028) solution for 15 min. The plaques were washed and counted for each plate, and plaque-forming units (PFU/mL) for each sample were determined.

### 2.4 Inhibitor, cytoxicity, and efficacy

The inhibitor used in this study was purchased from MedChemExpress, Omaveloxolone (OMA/RTA 408, Ca# HY-12212) and resuspended in DMSO (Fisher Chemical, Cat # 194474). HUVEC cells were seeded to reach ∼70–80% confluence overnight in white 96-well plates. For cytotoxicity, cells were treated for 24 h with OMA concentrations from 0.25 to 2 µM and compared to 2.5 µM DMSO, and cell viability assessed using the CellTiter-Glo® Luminescent Cell Viability Assay (Promega, Cat# G7573) per manufacturer’s instructions. For Efficacy, cells were pre-treated with OMA-containing media at 0.5 μM for 1 h, removed, and infected at a multiplicity of infection (MOI) of 0.1 with VEEV TC-83 luciferase tagged and relative luminescence assessed via Nano-Glo Luminescent Cell Viability Assay (Promega, Cat# G7573). Cytotoxicity and Maximum Efficacy Concentration for OMA were assessed in an 8-point dilution curve to determine the 50% cytotoxic concentration (CC_50_) and 90% maximum efficacy concentration (EC_90_) using GraphPad Prism 9.0 software. The concentration of OMA used in this study was within a non-toxic range (0.5 µM).

### 2.5 Immunofluorescence microscopy

HUVEC cells were seeded to reach ∼70–80% confluence into Cellvis 24-well glass bottom plates (Cat# P24-1.5H-N) and incubated overnight at 37 °C, 5% CO_2_. Cells were infected with VEEV TC-83 at an MOI of 1, 0.1, 0.01 or mock-infected with PBS. Following 1 h incubation, the inoculum was removed and replaced with fresh complete growth media. Infected cells were washed 3 times with PBS and fixed at 0-, 6-, 12-, 24-, and 48-hpi with 4% paraformaldehyde (Thermo, Cat# 28908) in 0.5% BSA (Thermo, Cat# 37520) for 10 min at room temperature (RT). Fixed cells were washed 3 times with PBS to remove fixation solution and permeabilized with 0.25% Triton X-100 (Fisher, Cat# X100-500 ML) in PBS for 5 min at RT. Fixed/permeabilized cells were washed 3 times with PBS and blocked with 3% BSA in PBS for 45 min at RT. Blocking solution was removed and cells incubated with primary antibodies: phalloidin Alex Fluor™ 488 at 1:600 (Thermo, Cat# A12379), VEEV E2 at 1:300 (Bioxcell, Cat# BE0435), and Hoechst 33342 at 1:800 (Cat#, R37165) in 3% BSA in PBS for 2 h at RT. After first incubation, the primary antibody solution was washed 3 times with PBS and cells were incubated with secondary antibody, donkey anti-mouse IgG (H + L) Alexa Fluor™ 568 (Thermo, Cat# A10037) for 1 h at RT. The secondary antibody solution was removed, and cells were washed 3 times with PBS and left in PBS for imaging. Images were acquired on the Discover Echo–CELLCYTE X™ using a ×10 objective.

### 2.6 RNA extraction

Cells were washed once with PBS (VWR, Cat# 24D1056367) after the collection of supernatants and lysed with RLT lysis buffer, and RNA extracted using the RNeasy 96 QIAcube HT Kit (Qiagen, Cat# 74171) and RNA extraction device from Qiagen following the manufacturer’s protocol. RNA yield and purity were quantified using the Thermo NanoDrop to ensure good quantity and quality of RNA extracted.

### 2.7 Library preparation

Library preparation was performed using the Zymo-Seq RiboFree Total RNA Library Kit (Zymo Research, Cat# R3000), following the manufacturer’s protocol. Sequencing was conducted on the Illumina NovaSeq X-Plus platform using a 25B flow cell with paired-end 150 bp reads (PE150). Each sample generated approximately 20 million read pairs, corresponding to about 6 Gb of raw data per sample, which provides typical depth for bulk transcriptome profiling. All libraries were sequenced with a read length of 150 bp per read in paired-end mode, which yields 300 bp of sequence per fragment and improves mapping across splice junctions and complex regions. For alignment, reads were mapped to the human reference genome (hg38) using hisat2 (version 2.2.1).

## 3 Methodology

All analyses were performed using five replicates per condition (n = 5 Mock, n = 5 OMA treated VEEV infected, n = 5 untreated VEEV infected). Raw sequencing reads were subjected to standard quality assessment, including evaluation of read length distributions, per base quality, adapter content, and duplication rates. To evaluate overall sample relatedness and confirm that replicates clustered according to their biological condition, a multidimensional scaling (MDS) (Saeed et al., 2018) analysis was performed.

### 3.1 Generalized linear model with Quasi-Likelihood and Magnitude-Altitude Scoring (GLMQL-MAS)

To investigate the transcriptomic responses during VEEV infection and the impact of OMA treatment, we employed the GLMQL-MAS framework (Rezapour et al., 2024a; Rezapour et al., 2024b; Rezapour et al., 2024c). This method is particularly suited for RNA-Seq count data due to its ability to account for overdispersion across biological replicates (Law et al., 2014). Two main contrasts were performed at 24 h post-infection (hpi):

- Untreated VEEV-infected samples (Unt) versus mock-infected controls,
- OMA-treated VEEV-infected samples (OMA) versus mock-infected controls.

This design allowed us to assess (i) the transcriptional shifts associated with VEEV infection in the absence of treatment and (ii) the modulatory effects of OMA during infection. Count normalization was performed using the Trimmed Mean of M-values (TMM) approach (Robinson and Oshlack, 2010) to correct for compositional differences between libraries. A design matrix was constructed to model the treatment and infection variables. For each gene, we fitted a negative binomial generalized linear model (GLM) (Nelder and Wedderburn, 1972), and quasi likelihood F tests (Wedderburn, 1974) were used to compare full and reduced models for each contrast. This GLMQL workflow in edgeR provided robust inference for differential expression while controlling mean-variance dependence in the counts. Gene-level dispersion estimates were obtained to capture inter-sample variability, which ensures robust inference. This approach enables rigorous detection of differentially expressed genes (DEGs) without relying on strict distributional assumptions. Multiple testing correction was performed using the Benjamini–Hochberg (BH) procedure (Benjamini et al., 2009; Benjamini and Hochberg, 1995), with a significance level of α=0.05 . To ensure biological relevance, we restricted our focus to genes with an absolute $\log_{2}$ fold change greater than 1 ( $\left|\text{LogFC}\right|>1,i.e.,\text{LogFC}>1\text{ or LogFC}<-1$ ).

*Significance thresholds:* In this study, a gene was considered significant if it satisfied both criteria: (i) a Benjamini–Hochberg (BH) adjusted p-value less than 0.05, and (ii) an absolute $\log_{2}$ fold change greater than 1. Hereafter, when we refer to a gene as “significant,” it is defined according to these criteria.

To further refine DEG prioritization, we incorporated the Magnitude-Altitude Scoring (MAS) method (Rezapour et al., 2024e; Rezapour et al., 2024f), which integrates both the magnitude of expression change and the statistical confidence. For each significant gene *l*, a composite MAS score was computed as shown in Equation 1:

$${\text{MAS}}_{l}=\left|\;\log_{2}({\text{FC}}_{l}{)|}^{M}\right|\log_{10}\left(p_{l}^{\text{BH}}\right){|}^{A}$$ (1)

where $p_{l}^{\text{BH}}$ denotes the BH adjusted p-value and ${\text{FC}}_{l}$ is the fold change. Parameters were set at M = A = 1 to balance the contributions of effect size and statistical strength. This scoring framework allowed us to systematically prioritize genes most strongly influenced by VEEV infection and OMA treatment, which highlights those with both robust regulation and statistical significance.

### 3.2 Functional and pathway enrichment analyses

Following the identification of differentially expressed genes, we focused our analyses on the significant upregulated genes, as these are more likely to represent host antiviral responses or treatment-induced protective pathways. In contrast, downregulated signals can often be indirect or reflect nonspecific suppression of cellular processes during infection. We conducted functional enrichment analyses to contextualize the upregulated DEGs. Gene Ontology (GO) enrichment (Catherine et al., 2000) was performed using the *clusterProfiler* R package (v4.14.6). Gene symbols were mapped to Entrez IDs with the *bitr()* function, and enrichment was carried out on Biological Process (BP) categories using *enrichGO()*. Enrichment results were filtered using BH correction, with a q-value <0.05 considered significant. In parallel, pathway enrichment analysis (Kanehisa and Goto, 2000) was conducted using the *GProfiler* Python API (*gprofiler-official*, v1.0.0), specifically querying curated Reactome (REAC) pathways to identify functional networks relevant to VEEV pathogenesis and OMA-mediated modulation.

### 3.3 Cross MAS to identify common and condition-specific genes

To identify genes that are shared between conditions and those that are condition specific, we applied the Cross-MAS ranking method (Algorithm 2 in (Rezapour et al., 2024d)) to the two GLMQL-MAS contrasts at 24 hpi: untreated VEEV versus mock and OMA treated VEEV versus mock. MAS scores and within contrast ranks were computed for all significant genes as defined above, and analyses focused on significant genes.

Let $S_{U}\left(g\right)\in \left\{0,1\right\}$ and $S_{\text{OMA}}\left(g\right)\in \left\{0,1\right\}$ indicate significance of gene *g* in the contrasts untreated VEEV vs. mock and OMA treated VEEV vs. mock, respectively. Let $r_{U}\left(g\right)$ and $r_{\text{OMA}}\left(g\right)$ be the corresponding MAS ranks (where 1 is the best). We define the Cross-MAS rank as shown in Equation 2:

$$R_{\text{CM}}\left(g\right)=\left\{\begin{array}{c} \max\left\{\;r_{U}\left(g\right),r_{\text{OMA}}\left(g\right)\;\right\},\\ r_{U}\left(g\right),\\ r_{\text{OMA}}\left(g\right),\\ \infty , \end{array}\right.\;\begin{array}{c} {\;\text{If}\;S}_{U}\left(g\right)=1\text{ and}{\;S}_{\text{OMA}}\left(g\right)=1,\\ {\;\text{If}\;S}_{U}\left(g\right)=1\text{ and}{\;S}_{\text{OMA}}\left(g\right)=0,\\ {\;\text{If}\;S}_{U}\left(g\right)=0\text{ and}{\;S}_{\text{OMA}}\left(g\right)=1,\\ {\;\text{If}\;S}_{U}\left(g\right)=0\text{ and}{\;S}_{\text{OMA}}\left(g\right)=0, \end{array}$$ (2)

where class assignment is defined as shown in Equation 3:

$$C\left(g\right)=\left\{\begin{array}{c} \text{common},\\ \text{unique to Untreated VEEV},\\ \text{unique to OMA treated VEEV},\\ \text{not selected}, \end{array}\right.\;\begin{array}{c} {\;\text{If}\;S}_{U}\left(g\right)=1\text{ and}{\;S}_{\text{OMA}}\left(g\right)=1,\\ {\;\text{If}\;S}_{U}\left(g\right)=1\text{ and}{\;S}_{\text{OMA}}\left(g\right)=0,\\ {\;\text{If}\;S}_{U}\left(g\right)=0\text{ and}{\;S}_{\text{OMA}}\left(g\right)=1,\\ {\;\text{If}\;S}_{U}\left(g\right)=0\text{ and}{\;S}_{\text{OMA}}\left(g\right)=0. \end{array}$$ (3)

Genes were then ordered in ascending rank, so that the gene with the lowest rank received the highest attention. For genes significant in both contrasts, the Cross-MAS rank is the maximum of the two ranks, which ensures strong performance in both conditions. For genes significant in only one contrast, the single available rank is used. This min-max approach highlights genes that are consistently strong across conditions while still capturing condition-specific signals.

To evaluate whether the top five upregulated and top five downregulated genes that are unique to OMA-treated VEEV and to untreated VEEV can separate sample classes, we performed hierarchical clustering. Counts were transformed as $\log_{2}$ (count +1) and then standardized per gene to Z scores across samples, which centers each gene at zero and scales to unit variance. Sample-to-sample distances were computed using Euclidean distance, and agglomeration was used with Ward linkage.

We also used the top-50 significantly upregulated genes that were unique to OMA-treated VEEV, unique to untreated VEEV, and common to both, which provided three gene sets for downstream analyses. First, we performed protein-protein interaction network analysis with STRING for Homo-sapiens, using the confidence score threshold of 0.5 and the confidence network flavor. Interactions were treated as undirected and weighted by STRING confidence. To reduce sparsity and emphasize highly connected genes, we removed self-loops and retained nodes with at least five degrees. Networks were examined separately for the OMA, unique, untreated unique, and common sets.

Second, we carried out independent functional enrichment for each of the three gene sets using the GProfiler Python client from the gprofiler-official package (v1.0.0) with organism set to human and with curated Reactome pathways. Results were ranked by the $\log_{10}$ (adjusted p-value) using the default multiple testing correction in GProfiler, and the top ten enriched terms were retained for reporting and visualization. This two-stage procedure allowed us to assess connectivity patterns among selected genes and to summarize their dominant pathway themes for OMA-treated, untreated, and common responses.

## 4 Results

**FIGURE 1:**
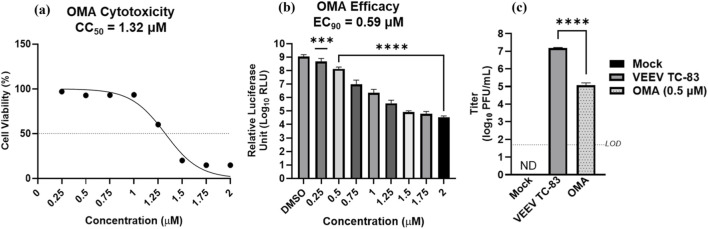
Evaluation of OMA cytotoxicity, efficacy, and impact on infectious viral load in HUVEC cells (n = 5). HUVEC cells were seeded to reach ∼70–80% confluence overnight. **(a)** Cells were treated with 8 (0.25–2 µM) concentrations of OMA compared to DMSO-treated cells at 2.5 µM (control), and viability was assessed via CellTiterGlo 24 h post-treatment. The dashed line indicates 50% viability. **(b)** Cells were pre-treated for 1 h with 0.5 µM of OMA or DMSO at 2.5µM, infected with luciferase-tagged VEEV TC-83 (MOI 0.1), and post-treated. Luminescence was assessed 24 h post-infection (hpi) using Nano-Glo. **(c)** Cells were pre-treated for 1 hour with 0.5 µM of OMA, infected with VEEV TC-83 (MOI 0.1), and post-treated or untreated (media only). Supernatants were collected 24 hpi, and infectious virus was quantified via plaque assay. The dashed line indicates the limit of detection (LOD) of 50 PFU/mL. Values are an average of five biological replicates ±standard deviation. ***p < 0.001, ****p < 0.0001. N.D. = not detected.

To determine the non-cytotoxic range of OMA in HUVEC cells, eight different concentrations were tested ranging from 0.25 to 2 µM (Figure 1a). The 50% cytotoxic concentration (CC_50_) was calculated to be 1.32 µM in these cells. The efficacy of OMA in HUVEC cells was also assessed within this range using a luciferase-tagged VEEV TC-83 (Figure 1b). Luciferase activity was significantly reduced at all concentrations and the effective concentration to achieve 90% inhibition was 0.59 µM. This correlates with the infectious titers calculated via plaque assay of supernatants collected 24 h post-infection (Figure 1c). Cells infected with VEEV TC-83 (MOI 0.1) yielded a titer of ∼7 log_10_ PFU/mL, while cells treated with 0.5 µM of OMA and infected at the same MOI, yielded a titer of ∼5 log_10_ PFU/mL or a >99.9% reduction in infectious viral load. Overall, the cytotoxicity and efficacy results validate OMA as a potential antiviral therapeutic.

**FIGURE 2:**
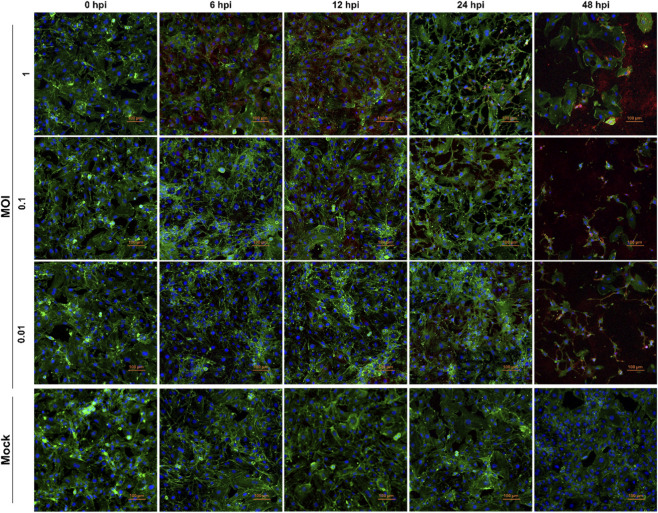
Impact of VEEV TC-83 infection in Human umbilical vein endothelial cells (HUVEC). HUVEC cells were seeded overnight to reach ∼70–80% confluence. Cells were infected with VEEV TC-83 at an MOI of 1, 0.1, 0.01 or mock-infected with PBS. Cells were then fixed and permeabilized at 0-, 6-, 12-, 24-, and 48-h post infection (hpi). Fixed cells were probed with F-actin/phalloidin (green), VEEV E2 (red), and DAPI (blue) at ×10 objective. Scale bar represents 100 µm and images are representative of three biological replicates.

A qualitative evaluation of viral infection and its progression in HUVEC cells was conducted using immunofluorescence microscopy. Cells were infected with VEEV TC-83 at an MOI of 1, 0.1, and 0.0, or mock-infected, and fixed at 0-, 6-, 12-, 24-, and 48-h post infection (Figure 2). Fixed cells were probed with fluorescent antibodies to visualize cell morphology/cytoskeleton, proliferation, and the presence or absence of viral proteins. There were no significant differences at 0 hpi between all conditions. However, changes in cell morphology were observed as early as 6 hpi in cells infected at an MOI of 1. These changes included cell shrinkage and long spindle-like projections between cells not observed in mock-infected cells. These changes in morphology were time-dependent where at an MOI of 0.1 changes were first observed at 12 hpi, whereas there was a 24-h delay (24 hpi) at an MOI of 0.01. In all infected conditions, the nuclear marker, DAPI, decreased in signal while the opposite was observed in mock-infected cells. Additionally, significant cell loss was observed in infected cells by 48 hpi. Altogether, these results provided evidence that the optimal MOI and time post infection for transcriptomic analysis was an MOI of 0.1 and 24 h post infection.

**FIGURE 3:**
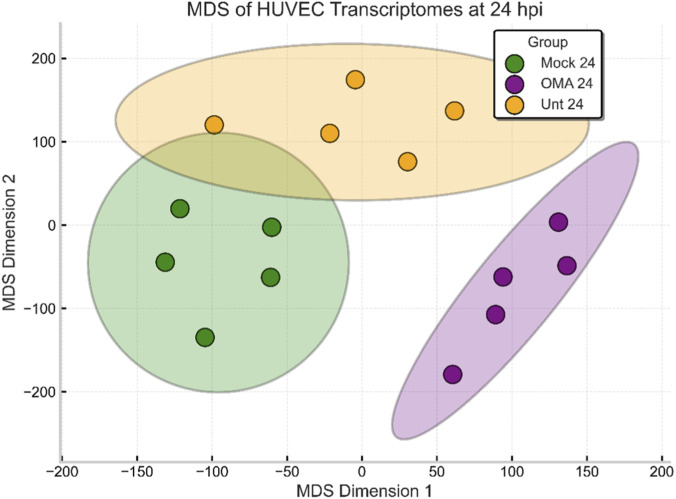
Multidimensional scaling (MDS) plot of HUVEC transcriptomes at 24 hpi. Each point represents one sample, and colors with shaded ellipses indicate Mock 24, OMA 24, and Unt 24 groups, which form distinct clusters in expression space.

Multidimensional scaling (MDS) (Saeed et al., 2018) analysis (Figure 3) demonstrated clear separation among the three experimental groups at 24 hpi. Mock, OMA treated, and untreated VEEV infected samples each formed distinct and nonoverlapping clusters in the two-dimensional MDS plane, which represents the pairwise transcriptional dissimilarity between samples. The tight grouping within each condition shows high consistency among biological replicates, and the pronounced separation between conditions confirms that the transcriptional profiles diverge according to infection status and OMA treatment.

### 4.1 Generalized linear model with Quasi-Likelihood and Magnitude-Altitude Scoring (GLMQL-MAS)

**FIGURE 4:**
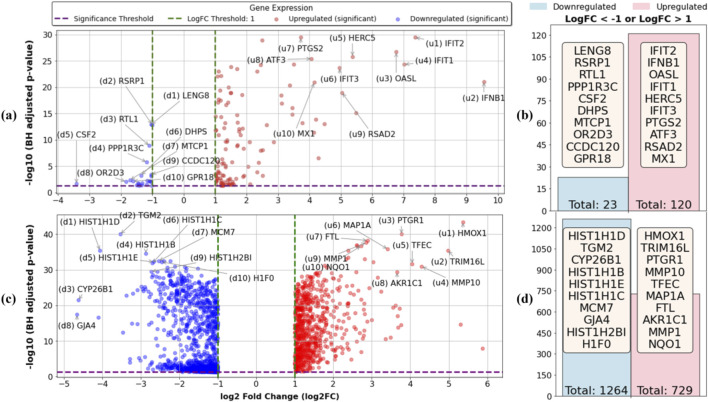
Transcriptomic responses to VEEV infection and OMA treatment at 24 hpi. **(a)** Volcano plot showing differentially expressed genes (DEGs) between untreated VEEV-infected and mock-infected samples, with the top 10 upregulated and downregulated genes annotated. Only significant genes are displayed. Note that u1-u10 denote the top-ranked upregulated genes and d1-d10 represent the top-ranked downregulated genes. Due to marker size, some points may appear on logFC = 1 or −1, though the actual thresholds are logFC >1 and logFC < −1. **(b)** Summary of total upregulated and downregulated DEGs for the same contrast. **(c)** Volcano plot showing DEGs between OMA-treated VEEV-infected and mock-infected samples, with the top 10 upregulated and downregulated genes annotated. **(d)** Summary of total upregulated and downregulated DEGs for the OMA-treated contrast.

To characterize the transcriptomic shifts induced by VEEV infection and OMA treatment, we applied the GLMQL-MAS framework to identify differentially expressed genes across the experimental contrasts. Dispersion estimates showed the expected mean–variance relationships for RNA-Seq count data in both contrasts, which supports the use of the quasi-likelihood framework (Supplementary Figures S1, S2). Figure 4 summarizes the differentially expressed genes (DEGs) identified using GLMQL-MAS analysis across the two main contrasts. Figure 4a shows the volcano plot comparing untreated VEEV-infected samples against mock-infected controls at 24 hpi. Only significant genes are displayed for clarity, with the top 10 upregulated and downregulated genes annotated. Among the strongly upregulated genes were well-characterized antiviral effectors, including *IFIT1*, *IFIT2*, *IFIT3*, *OASL*, *RSAD2*, and *MX1*. These genes represent classic interferon-stimulated responses to viral infection. In contrast, several host regulatory genes such as *RSRP1*, *DHPS*, and *PPP1R3C* were downregulated.

Figure 4b quantifies the total number of significant DEGs from this contrast, revealing 120 significantly upregulated and 23 significantly downregulated genes. Figure 4c presents the volcano plot comparing OMA-treated VEEV-infected samples against mock-infected controls at 24 hpi, again showing only significant genes for clarity and annotating the top 10 up- and downregulated transcripts. Here, strong induction of genes linked to stress response and redox regulation was observed, including *HMOX1*, *NQO1*, *FTL*, and *AKR1C1*, alongside extracellular matrix-related genes such as *MMP1* and *MMP10*. Conversely, multiple histone cluster genes (*HIST1H1B*, *HIST1H1D*, *HIST1H2BI*, etc.) were markedly downregulated, which suggests epigenetic remodeling under OMA treatment. Figure 4d summarizes the global DEG counts for this contrast, identifying 729 upregulated and 1,264 downregulated genes. Compared to untreated infection, OMA treatment produced a more extensive transcriptomic shift, characterized by both strong induction of antioxidant and metabolic regulators and widespread repression of chromatin-associated transcripts.

### 4.2 Functional and pathway enrichment analyses

**FIGURE 5:**
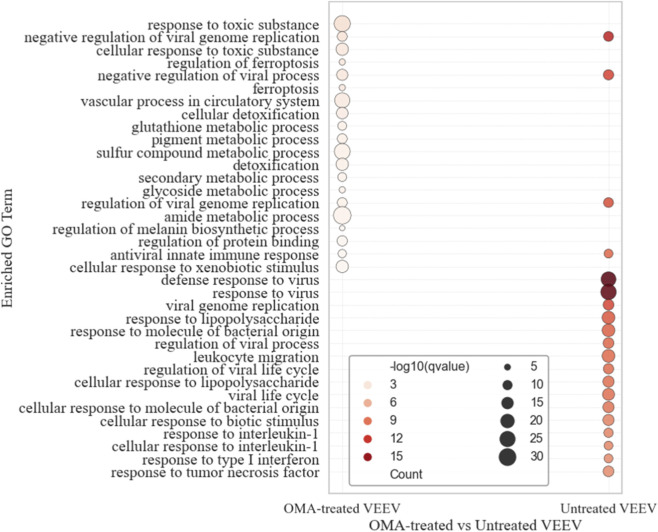
Gene Ontology (GO) enrichment analysis of upregulated genes. Bubble plot showing enriched Biological Process (BP) terms for significant upregulated genes at 24 hpi. Circle size corresponds to the number of genes within each GO category, and color intensity reflects enrichment significance (-log10 q-value). Results are displayed for OMA-treated VEEV-infected samples versus mock, and untreated VEEV-infected samples versus mock.

To place the differentially expressed genes into a biological context, we next examined their functional enrichment. Figure 5 shows the enriched GO Biological Process terms associated with significant upregulated genes for both untreated and OMA-treated VEEV-infected samples at 24 hpi. In the untreated condition, enriched terms were largely immune-related, including “antiviral responses,” “interferon signaling,” “cytokine responses,” “leukocyte migration,” and “regulation of viral replication and life cycle.” These enrichments reflect strong activation of host defense and immune regulatory pathways during infection.

In contrast, the OMA-treated condition showed enrichment of processes related to metabolism, detoxification, and cellular stress responses, such as “glutathione metabolism,” “ferroptosis,” “sulfur compound metabolism,” “cellular detoxification,” and “xenobiotic responses,” in addition to “vascular” and “pigment metabolic processes.”

These categories highlight a shift toward stress-adaptive and metabolic reprogramming pathways under OMA treatment. Despite these differences, both conditions shared a small set of common antiviral categories, including “antiviral innate immune response” and “negative regulation of viral genome replication.”

**FIGURE 6:**
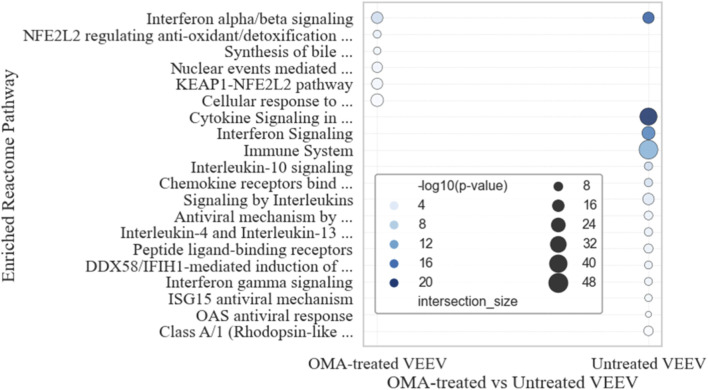
Reactome pathway enrichment analysis of upregulated genes. Bubble plot showing enriched Reactome pathways for significant upregulated genes at 24 hpi. Circle size represents the number of enriched genes per pathway, and color intensity reflects enrichment significance (-log10 p-value). Results are shown for OMA-treated VEEV-infected samples versus mock, and untreated VEEV-infected samples versus mock.

Figure 6 shows the Reactome pathway enrichment results for significant upregulated genes at 24 hpi across infection conditions. In the untreated VEEV-infected samples versus mock, enriched pathways were primarily immune-related, including “interferon signaling (alpha/beta, gamma),” “interleukin signaling (IL-4, IL-10, IL-13),” “ISG15 antiviral mechanism,” “OAS antiviral response,” “chemokine receptor signaling,” “cytokine signaling in the immune system,” and “*DDX58*/*IFIH1*-mediated induction of interferon-alpha/beta.”

For the OMA-treated VEEV-infected samples versus mock, enriched pathways were distinct and clustered around cellular stress and detoxification processes. These included “KEAP1-NFE2L2 pathway,” “NFE2L2 regulating antioxidant/detoxification enzymes,” “nuclear events mediated by NFE2L2,” “cellular response to chemical stress,” and “synthesis of bile acids and bile salts via 27-hydroxycholesterol.”

In addition to examining upregulated transcripts, we evaluated pathway enrichment for significantly downregulated genes in both contrasts. Under the stringent significance criteria defined for this study (BH adjusted p < 0.05 and |log_2_FC| > 1), the untreated VEEV condition produced a small set of downregulated genes that did not yield any Reactome pathways passing multiple testing correction, which indicates that these decreases did not form a coherent or interpretable pathway-level signature. In contrast, OMA treated VEEV samples showed a large number of significantly downregulated genes that mapped to cell cycle and chromatin-associated pathways, which include “Cell Cycle,” “Cell Cycle, Mitotic,” “Cell Cycle Checkpoints,” “Mitotic Spindle Checkpoint,” “G1/S Transition,” and “DNA Replication.” These categories reflect broad suppression of proliferative programs rather than targeted modulation of antiviral or immune mechanisms (see Supplementary Figure S3).

### 4.3 Cross MAS to identify common and condition specific genes

**FIGURE 7:**
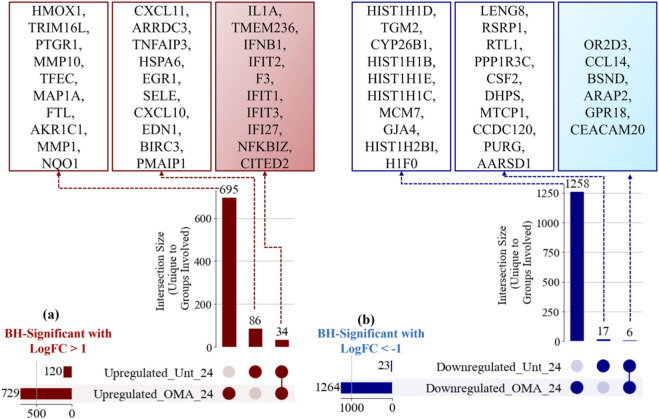
Cross-MAS partition of significant genes at 24 hpi. **(a)** Significant upregulated genes with totals per condition, counts for genes unique to OMA treated, unique to untreated, and common, with the top ten genes for each group annotated. **(b)** Significant downregulated genes with totals per condition, counts for genes unique to OMA treated, unique to untreated, and common, with the top ten genes for each group annotated.

While enrichment analyses describe broad pathway-level shifts, it is also important to determine which genes are unique to each condition and which are shared. Figure 7 reports the Cross-MAS analysis at 24 hpi. For significant upregulated genes, OMA treated VEEV showed 729 genes and untreated VEEV showed 120 genes. The partition yielded 695 genes unique to OMA treated, 86 genes unique to untreated, and 34 genes common to both sets. The top ten genes in each group based on Cross-MAS ranking are listed. For significant downregulated genes, OMA treated VEEV showed 1,264 genes and untreated VEEV showed 23 genes. The partition yielded 1,258 genes unique to OMA treated, 6 genes unique to untreated, and 17 genes common to both sets. The top ten genes in each group based on Cross MAS ranking are annotated.

**FIGURE 8:**
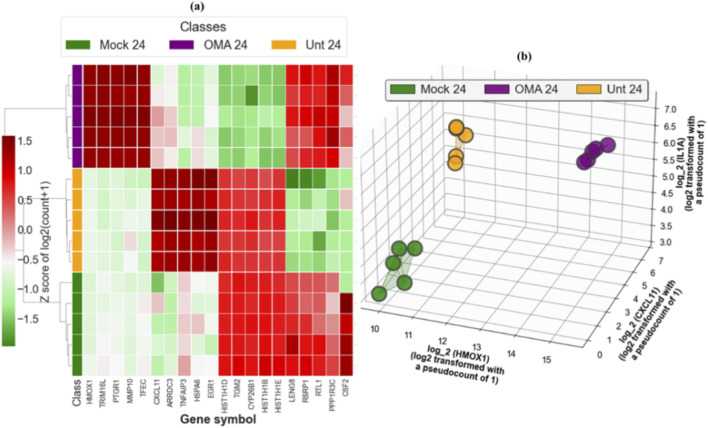
Separation of samples by selected genes. **(a)** Hierarchical clustering heat map of Z scores of log2 (count +1) for the top five downregulated genes unique to OMA treated VEEV and the top five downregulated genes unique to untreated VEEV, with rows clustered and columns fixed by gene order. **(b)** Three-dimensional scatter of log2 (count +1) for HMOX1, CXCL11, and IL1A, one axis per gene.

Figure 8 displays separation of samples based on selected genes. Figure 8a shows a heat map with hierarchical clustering of the top five upregulated and downregulated genes unique to OMA treated VEEV and the top five upregulated and downregulated genes unique to untreated VEEV, which separates mock, OMA treated, and untreated samples into distinct clusters. Figure 8b shows a three-dimensional scatter using the log transformed values of three genes, *HMOX1*, *CXCL11*, and *IL1A*, which are the top upregulated genes selected by Cross-MAS that are unique to OMA, unique to untreated, and common to both, respectively.

### 4.4 Network and enrichment analyses of condition-specific genes

**FIGURE 9:**
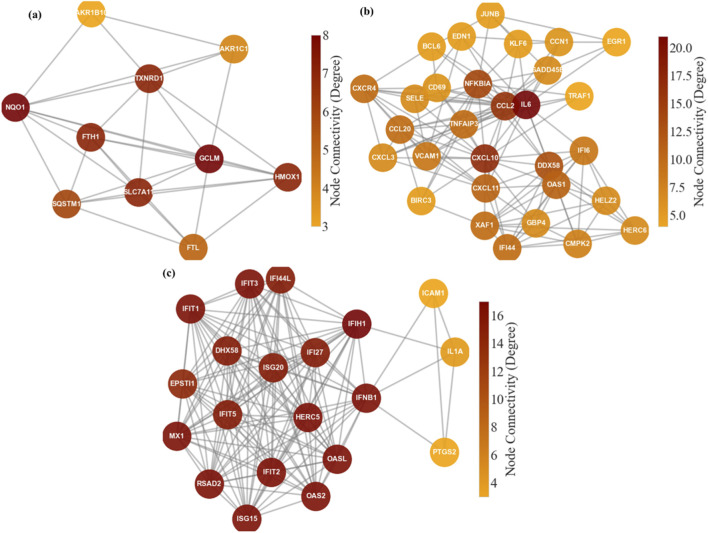
Network structure of OMA unique, untreated VEEV unique, and common gene sets. **(a)** STRING network for OMA unique genes, which forms a single NRF2 centered module with hubs including NQO1, GCLM, HMOX1, TXNRD1, FTH1, and SLC7A11. **(b)** STRING network for untreated VEEV unique genes, which separates into a cytokine/chemokine cluster (for example IL6, CXCL10, CXCL11, CCL2) and an interferon/ISG cluster (for example DDX58, OAS1, IFI44, IFI6). **(c)** STRING network for genes common to both conditions, which forms a dense antiviral module centered on interferon signaling and ISGs, including IFIT1–3, OAS2, OASL, ISG15, RSAD2, MX1, IFIH1, and IFNB1.

Beyond cataloging individual genes, we sought to understand how condition-specific responses organize into networks. Protein–protein interaction analyses and Reactome enrichment of top-ranked gene sets revealed distinct NRF2-centered and cytokine-centered modules for OMA-treated versus untreated infection. Figure 9 compares the STRING network structure for OMA unique, untreated VEEV unique, and common gene sets. In the OMA specific set (Figure 9a), 26 of 50 submitted genes mapped to STRING, which produced 60 interactions and two components before filtering. Applying the degree threshold retained 10 nodes and 31 edges, which formed one dense community with average degree 6.2, average shortest path length 1.33, and clustering coefficient 0.54. The highest degree nodes were *NQO1* and *GCLM* (degree 8), followed by *HMOX1*, *TXNRD1*, *FTH1*, and *SLC7A11* (degree 7) and *SQSTM1* (degree 6), with the strongest edges between *FTL–FTH1* (score 0.999), *NQO1–GCLM* (0.949), *HMOX1–NQO1* (0.916), *HMOX1–GCLM* (0.910), and *NQO1–TXNRD1* (0.803).

The untreated VEEV specific network (Figure 9b) was larger and less dense: 37 of 50 queries mapped, and after degree filtering, 30 nodes and 133 edges formed a single high connectivity component with density 0.306, average degree 8.87, average clustering 0.50, and mean shortest path 1.87. Greedy modularity identified two communities, a cytokine and chemokine cluster containing *IL6*, *CXCL10*, *CXCL11*, *CCL2*, *CCL20*, *CXCR4*, *VCAM1*, *SELE*, *NFKBIA*, *TNFAIP3*, *TRAF1* and a type I interferon and ISG cluster containing *DDX58*, *OAS1*, *IFI44*, *IFI6*, *XAF1*, *HERC6*, *GBP4*, *HELZ2*, *CMPK2*. Hubs by degree and weighted degree included *IL6*, *CXCL10*, *CCL2*, *NFKBIA*, and *DDX58*, with high confidence edges such as *CXCL10–CXCL11*, *CCL2–CXCR4*, *CCL20–CXCL11*, *TRAF1–BIRC3*, and *IL6–CXCL10*.

In contrast, the common gene set (Figure 9c) formed the most compact network: of 34 submitted genes, 26 mapped, and after degree filtering the network contained 20 nodes and 138 edges with high density 0.726, average degree 13.8, average shortest path 1.31, and average clustering 0.79. Two communities were detected, a larger antiviral module containing *IFIT1*, *IFIT2*, *IFIT3*, *OAS2*, *OASL*, *ISG15*, *RSAD2*, *MX1*, *HERC5*, *IFI27*, *IFI44L*, *EPSTI1* and a smaller group containing *IFIH1*, *IFNB1*, *IL1A*, *PTGS2*, and *ICAM1*. *IFIH1* showed the highest degree, whereas *IFNB1* showed the highest betweenness, which emphasizes the central role of this shared interferon module in both conditions.

**FIGURE 10:**
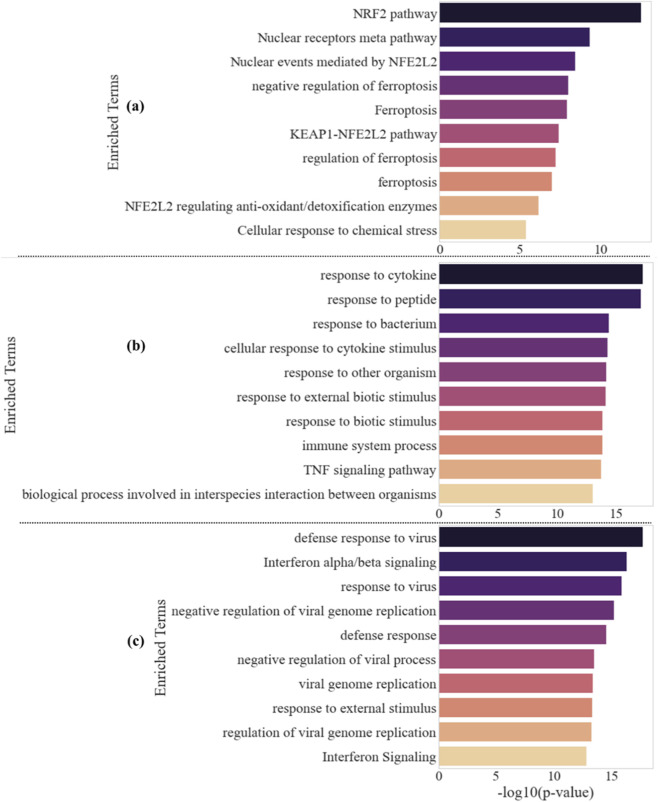
Pathway enrichment for OMA unique, untreated VEEV unique, and common gene sets. **(a)** Top ten enriched pathways and processes for genes unique to OMA treated VEEV ranked by -log10 p value, highlighting NRF2 centered redox, detoxification, and ferroptosis related terms. **(b)** Top ten enriched terms for genes unique to untreated VEEV infection, dominated by cytokine, inflammatory, and TNF signaling categories. **(c)** Top ten enriched terms for genes shared between OMA treated and untreated VEEV, defining a conserved interferon driven antiviral backbone that includes defense response to virus and interferon alpha beta signaling.

Figure 10 compares pathway level enrichment across OMA unique, untreated VEEV unique, and common gene sets. Figure 10a shows the top ten enrichment terms for genes unique to OMA treated VEEV ranked by -log_10_ *p* value. The leading terms are “NRF2 pathway” and “Nuclear receptors meta pathway,” followed by Reactome “Nuclear events mediated by NFE2L2,” GO “negative regulation of ferroptosis,” “Ferroptosis,” Reactome “KEAP1 NFE2L2 pathway,” GO “regulation of ferroptosis,” GO “ferroptosis,” Reactome “NFE2L2 regulating anti-oxidant and detoxification enzymes,” and Reactome “Cellular response to chemical stress.” This profile is consistent with a KEAP1–NRF2 axis that enhances redox control, detoxification, and ferroptosis restraint under OMA treatment.

Figure 10b shows the top ten enriched terms for genes unique to untreated VEEV infection. The dominant categories are “response to cytokine,” “response to peptide,” “response to bacterium,” “cellular response to cytokine stimulus,” “immune system process,” and the KEGG “TNF signaling pathway,” which indicate a strongly cytokine driven, inflammatory, and broadly innate immune state in the absence of OMA. Figure 10c shows the top ten enriched terms for genes shared between OMA treated and untreated VEEV. The leading categories are “defense response to virus,” “interferon alpha beta signaling,” “response to virus,” “negative regulation of viral genome replication,” “defense response,” “negative regulation of viral process,” “viral genome replication,” “response to external stimulus,” “regulation of viral genome replication,” and “interferon signaling,” which together define a conserved interferon centered antiviral backbone that remains engaged regardless of treatment.

## 5 Discussion

This study reveals two distinct transcriptional landscapes: an interferon and cytokine driven program under untreated VEEV infection, and an NRF2 centered cytoprotective program under OMA treatment. These findings extend our prior antiviral studies in which OMA reduced VEEV and EEEV burden in multiple cell types and preserved vascular and neural barrier properties in a human NVU organ-chip model while limiting inflammatory cytokines (Boghdeh et al., 2023; Boghdeh et al., 2022). This background established the rationale for transcriptional profiling in primary human endothelial cells.

Because NRF2 signaling, lipid peroxidation, and ferroptosis were not directly measured here, the NRF2 centered interpretation remains a mechanistic hypothesis supported by our previous studies. Our earlier TC-83 studies showed that VEEV infection drives ROS accumulation, mitochondrial depolarization, PINK1–Parkin recruitment, mitophagy, and redox-dependent cytokine production in CNS cells, while mitochondria targeted antioxidants restored membrane potential, lowered ROS, and reduced caspase activity and cytokine release (Keck et al., 2018; Keck et al., 2017). Together with the antiviral and anti-inflammatory effects of OMA in conventional cultures and NVU platforms (Boghdeh et al., 2023; Boghdeh et al., 2022), these findings support the biological plausibility of the NRF2 and ferroptosis related signatures observed here, while highlighting the need for functional validation in endothelial cells.

Transcriptome analysis of this study showed that untreated infection induces a strong ISG signature, including *IFIT1–IFIT3*, *OASL*, *RSAD2*, and *MX1*, consistent with canonical antiviral programs. OMA treatment, by contrast, induced antioxidant and stress-response genes such as *HMOX1*, *NQO1*, *FTL*, and *AKR1C1* and suppressed histone clusters, indicating broader metabolic and chromatin reorganization. These features suggest that OMA shifts the host away from a purely interferon driven profile toward an NRF2 linked stress adaptive state that may limit viral propagation.

Pathway analyses reinforced these distinctions. Untreated VEEV activated inflammatory programs, including “cellular response to interleukin-1,” “defense response to virus,” “leukocyte migration,” and “Interferon gamma signaling,” alongside pattern recognition and cytokine pathways such as “response to lipopolysaccharide,” “response to tumor necrosis factor,” and “ISG15 antiviral mechanism.” This pattern aligns with PRR-driven IRF and NF-κB activation during alphavirus infection (Schoggins and Rice, 2011). In contrast, OMA enriched for KEAP1–NFE2L2 axis pathways “KEAP1–NFE2L2 pathway,” “NFE2L2 regulating antioxidant/detoxification enzymes,” and “nuclear events mediated by NFE2L2,” which are hallmarks of NRF2 mediated cytoprotection (Yamamoto et al., 2018). These categories include detoxification, iron regulation, and lipid peroxide control, consistent with NRF2’s ability to dampen excessive cytokine signaling while maintaining baseline antiviral restriction. The absence of “viral life cycle” and “viral genome replication” terms in OMA treated cells may further reflect suppression of virus driven transcriptional programs.

Cross-MAS analysis showed that OMA elicits a far broader transcriptional shift than infection alone, with a small, shared core and a dominant OMA specific program. Hierarchical clustering and minimal-marker projection using *HMOX1*, *CXCL11*, and *IL1A* cleanly separated mock, OMA treated, and untreated groups, confirming an NRF2 centered OMA signature versus a chemokine dominated untreated signature.

The network architecture further emphasizes these distinctions. The OMA specific module forms a single coherent NRF2 program dominated by *NQO1*, *GCLM*, *TXNRD1*, *SLC7A11*, and *HMOX1*. These genes support quinone reduction, glutathione synthesis, thioredoxin recycling, and protection from lipid peroxidation (Zanotto-Filho et al., 2016; Koppula et al., 2021; Kerins and Ooi, 2018). Ferritin subunits *FTH1* and *FTL* cluster with *HMOX1* and align with NRF2 linked iron sequestration that lowers the labile iron pool (Kerins and Ooi, 2018). *SQSTM1* connects selective autophagy with NRF2 through the p62–Keap1 axis (Ichimura and Komatsu, 2018; Katsuragi et al., 2016; Kage et al., 2020). This structure supports the interpretation that OMA concentrates the response into an NRF2 centered cytoprotective module that strengthens redox control and limits ferroptotic pressure (Shakya et al., 2023; Dong et al., 2021).

The untreated infection network is instead dominated by inflammatory and antiviral signaling. Nodes such as *IL6*, *CCL2*, *CXCL10*, *CXCL11*, *CXCR4*, *VCAM1*, and *SELE* indicate endothelial activation and leukocyte recruitment (Katkenov et al., 2024; Zhang et al., 2024). A connected antiviral cluster links *DDX58* with ISGs such as *OAS1*, *IFI44*, *IFI6*, and *XAF1*, supporting type I interferon mediated restriction (Rehwinkel and Gack, 2020). Negative regulators *NFKBIA* and *TNFAIP3* appear among central nodes, consistent with feedback control of NF-κB signaling (Rothschild et al., 2018). The shared network reflects a conserved antiviral backbone driven by *IFIH1*, *DHX58*, *IFNB1*, and ISGs including *IFIT1– IFIT3*, *OAS2*, *OASL*, *MX1*, *RSAD2*, and *ISG15*, which propagate interferon signaling and antiviral effector functions (Song et al., 2015).

Pathway enrichment profiles parallel these structural differences. OMA specific terms including “NRF2 pathway,” “KEAP1–NFE2L2 pathway,” multiple ferroptosis related categories, and detoxification modules, reflect NRF2 controlled redox and iron homeostasis (Tonelli et al., 2018; Reisman et al., 2019; Tang et al., 2021; Anandhan et al., 2020; Hayes et al., 2009). Untreated infection enriched for cytokine and immune signaling pathways such as “response to cytokine,” “immune system process,” and “TNF signaling pathway,” consistent with the action of *IL6*, *CCL2*, *CXCL10*, *CXCL11*, *CCL20*, *CXCR4*, *VCAM1*, and *SELE* during endothelial inflammatory activation (riedrichs et al., 1998; Wyble et al., 1997; Shen et al., 1997). The common enrichment set highlighted “defense response to virus,” “interferon alpha/beta signaling,” and “negative regulation of viral genome replication,” with effector arms such as the *IFIT* family and OAS–RNase L axis represented through *IFIT1–5*, *OAS2*, and *OASL* (Ivashkiv and Donlin, 2014; Fleith et al., 2018; Drappier and Michiels, 2015; Kristiansen et al., 2010).

Together, these results show that OMA rebalances the endothelial response to VEEV infection by redirecting transcriptional output from an interferon and cytokine dominated inflammatory state toward an NRF2 centered cytoprotective program, while preserving core antiviral defenses. These findings suggest that NRF2 activation may offer a strategy to limit VEEV induced endothelial dysfunction by enhancing redox control and reducing inflammatory damage without suppressing essential antiviral pathways. Future work should directly assess NRF2 activation, lipid peroxidation, ferroptotic sensitivity, and iron handling in OMA treated endothelial cells, and should evaluate whether combining NRF2 directed approaches with antiviral drugs provides additive or synergistic protection in advanced human NVU models and *in vivo* systems.

### 5.1 Limitations of the study

The study is conducted in primary human umbilical vein endothelial cells (HUVECs). While HUVECs are a well-accepted model for vascular biology, they may not fully capture the diversity of endothelial responses across different vascular beds (e.g., brain microvascular endothelium, pulmonary endothelium) that are highly relevant for VEEV neurotropism. Moreover, the study uses the attenuated VEEV TC-83 strain, not a wild-type or highly pathogenic strain. Transcriptomic responses to TC-83 may differ from those elicited by virulent epizootic strains, which could limit translation of findings to natural infection.

Another limitation is that our experimental design did not include an OMA only, uninfected control group. As a result, we cannot fully separate transcriptional changes that are driven exclusively by OMA in resting endothelial cells from those that are specific to the combination of OMA treatment and VEEV infection. Although our current comparisons between VEEV infected cells with and without OMA highlight how NRF2 activation reshapes the infected state, they do not capture the complete spectrum of OMA effects in the absence of infection. Future studies will incorporate a full 2 × 2 design, which will directly compare mock and VEEV infected HUVECs treated with vehicle or OMA and will allow a more precise dissection of drug specific, infection specific, and interaction driven responses.

Moreover, this study focuses on transcriptional responses at 24 h post infection, which provides a clear snapshot of pathway level changes but does not capture the temporal dynamics of NRF2 activation or interferon signaling. Earlier and later time points, such as 6 h and 48 h post infection, would allow a more detailed assessment of the kinetics of antiviral and stress response pathways and may reveal transient or delayed regulatory events that are not detectable at 24 h. Inclusion of these additional time points represents an important direction for future work and will help further clarify how OMA influences the progression of VEEV induced cellular responses over time.

Finally, although OMA is an FDA-approved drug, its antiviral potential remains speculative in the absence of *in vivo* infection models. Thus, the present work should be viewed as hypothesis-generating, laying the groundwork for future studies in diverse endothelial and neuronal models, across multiple time points, and ultimately in animal systems that better recapitulate pathogenic infection.

## 6 Conclusion

This study provides a detailed transcriptomic dissection of Venezuelan equine encephalitis virus (VEEV) infection and its modulation by omaveloxolone (OMA) treatment. By applying the GLMQL-MAS framework with Cross-MAS prioritization, we systematically identified condition-specific and shared transcriptional programs, which revealed distinct molecular signatures that separate untreated infection, OMA-treated infection, and mock controls.

Untreated VEEV infection was defined by robust activation of interferon-stimulated genes (ISGs), including *IFIT1*, *IFIT2*, *IFIT3*, *OASL*, *RSAD2*, and *MX1*, alongside proinflammatory cytokines and chemokines such as *IL6*, *CXCL10*, and *CXCL11*. Enrichment analyses confirmed a strong induction of interferon signaling, cytokine signaling, leukocyte migration, and viral defense pathways. The untreated condition also demonstrated heightened responses to bacterial-like molecular signals and tumor necrosis factor (TNF) and interleukin pathways, which indicates a broad inflammatory and antiviral state dominated by cytokine and interferon signaling. Protein interaction networks placed *IL6*, *CXCL10*, *CCL2*, and *DDX58* as central hubs, which reflects tightly interconnected cytokine-chemokine and antiviral sensing modules.

In contrast, OMA treatment produced a far more extensive and distinct transcriptional program, with 729 upregulated and 1,264 downregulated genes compared to 120 and 23, respectively, in untreated infection. Prominent OMA-induced genes included *HMOX1*, *NQO1*, *GCLM*, *TXNRD1*, *FTL*, *FTH1*, *AKR1C1*, *SLC7A11*, and *SQSTM1*. These are canonical NRF2-driven targets linked to antioxidant defense, ferroptosis resistance, glutathione synthesis, thioredoxin recycling, iron metabolism, and selective autophagy. Concurrent downregulation of histone cluster genes (*HIST1H1B*, *HIST1H1D*, *HIST1H2BI*, etc.) suggested epigenetic reprogramming under OMA. Enrichment profiles highlighted NRF2-centered pathways, including KEAP1-NFE2L2 signaling, ferroptosis regulation, cellular detoxification, and xenobiotic metabolism, supporting a cytoprotective and redox-balancing transcriptional state distinct from untreated infection.

Cross-MAS analysis demonstrated that OMA treatment yielded 695 unique upregulated genes and 1,258 unique downregulated genes, whereas untreated infection contributed only 86 unique upregulated and 6 unique downregulated genes. A small but important core of 34 upregulated and 17 downregulated genes was shared, which represents a conserved antiviral backbone. Hierarchical clustering of the top-ranked unique genes cleanly separated all conditions, while a minimal marker set (*HMOX1*, *CXCL11*, *IL1A*) was sufficient to distinguish OMA-treated, untreated, and mock samples, underscoring distinct molecular states.

Network-level analyses revealed that OMA responses were organized around a dense antioxidant and detoxification module, in which *NQO1*, *GCLM*, *TXNRD1*, *SLC7A11*, and *HMOX1* functioned as hubs, linking ferroptosis resistance to glutathione and iron metabolism. In contrast, untreated infection networks were dominated by inflammatory mediators (*IL6*, *CXCL10*, *CCL2*, *VCAM1*, *SELE*) and antiviral sensors (*DDX58*, *OAS1*, *IFI44*, *IFI6*), which reflect strong cytokine and interferon-driven immunity. The common set preserved a compact antiviral hub, with *IFIH1*, *DHX58*, *IFNB1*, *IFIT* family members, *OAS2*, *OASL*, *RSAD2*, *MX1*, and *ISG15* forming a tightly interconnected interferon-centered module that maintains direct restriction of viral genome replication and translation.

Collectively, these findings demonstrate that untreated VEEV infection relies primarily on a proinflammatory, interferon-centric program to combat viral replication. In contrast, OMA treatment reprograms the host transcriptome toward NRF2-driven antioxidant defense, ferroptosis suppression, detoxification, and chromatin remodeling. Despite this divergence, both conditions preserve a conserved antiviral core that ensures baseline restriction of viral replication. This work reveals that OMA expands host defenses beyond canonical interferon signaling, establishing a distinct cytoprotective and stress-adaptive antiviral state that may reduce immunopathology while maintaining antiviral restriction.

## Data Availability

The datasets presented in this article are not readily available because the current study is part of the Host signaling mechanisms contributing to endothelial damage in hemorrhagic fever virus infection, funded by the US government. In recognition of the significance of data sharing and transparency in advancing scientific research, we understand the interest in accessing these datasets. However, due to specific governance and the sensitive nature of the data, these datasets are not publicly available. Researchers who satisfy the established criteria for data access can request the data. These criteria are designed to ensure that data sharing is conducted responsibly and in a manner that respects the guidelines. Requests to access the datasets should be directed to Mostafa Rezapour (Mostafa.Rezapour@advocatehealth.org). Each request will be reviewed promptly, and access will be granted where it is deemed reasonable and permissible under our agreements. We assure prospective researchers that all requests will be considered carefully, with the aim of fostering scientific collaboration and adhering to the principles of transparency and data sharing.
