# Supplementary material for: Transcriptomic profiling of human endothelial cells infected with venezuelan equine encephalitis virus reveals NRF2 driven host reprogramming mediated by omaveloxolone treatment
Source: Front Genet. 2025 Dec 18;16:1722527. doi: 10.3389/fgene.2025.1722527 (PMC12755857; doi:10.3389/fgene.2025.1722527)
Supplement: Supplementary file 1 [file DataSheet1.docx]

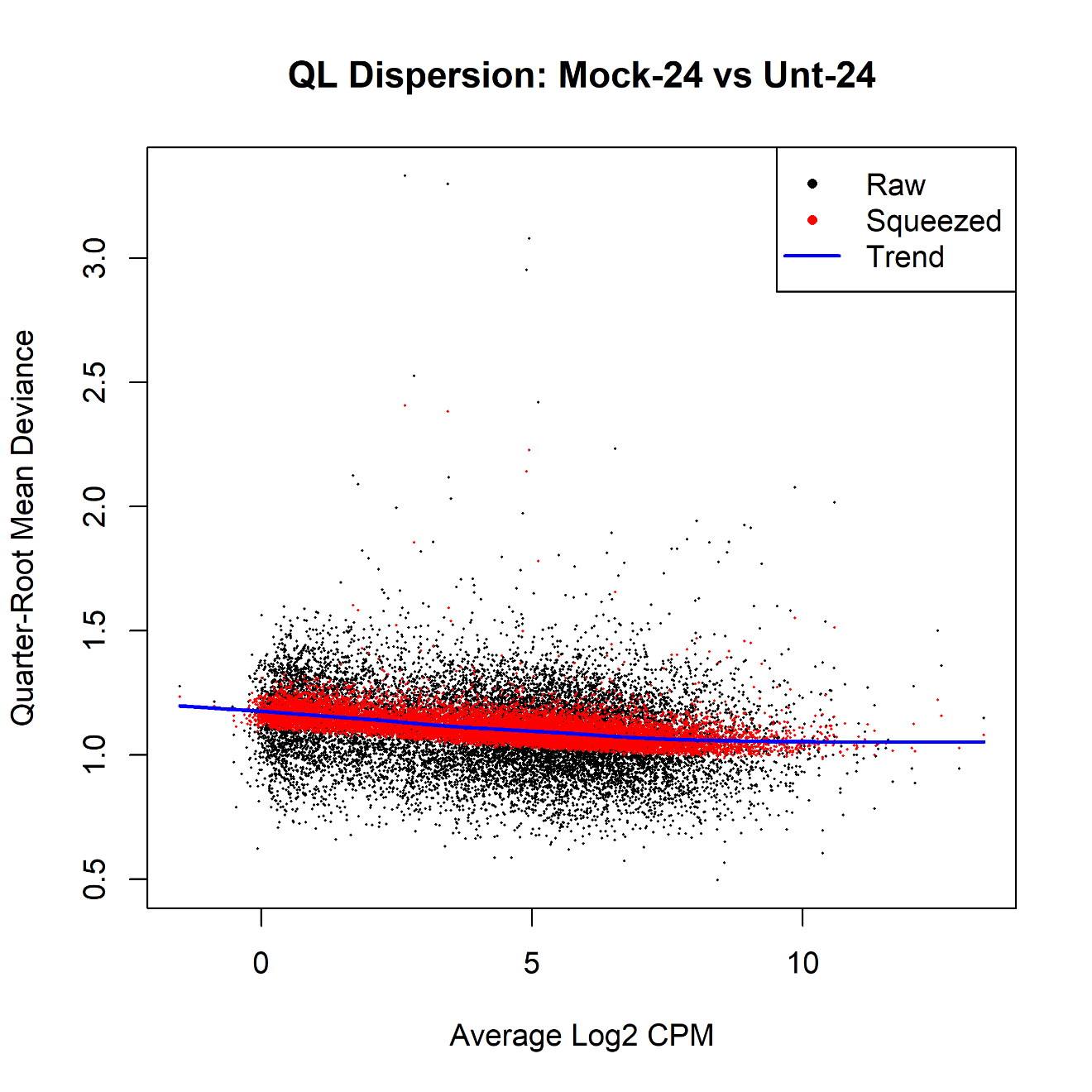


**Figure S1. Quasi-likelihood diagnostics for untreated VEEV versus mock-infected at 24 hpi.** Quarter-root mean deviance from the edgeR GLM quasi-likelihood analysis comparing untreated VEEV TC-83 infected HUVEC samples (Unt-24, n = 5) to mock-infected controls (Mock-24, n = 5). Each point represents one gene, the x-axis shows the average log₂ counts per million (logCPM), and the y-axis shows the quarter-root mean deviance. Black points indicate raw gene-wise deviances, red points indicate squeezed (shrunken) deviances, and the blue line represents the fitted mean–variance trend. The smooth, gently decreasing trend with limited high-deviance outliers indicates that dispersion and deviance are well controlled for this contrast.


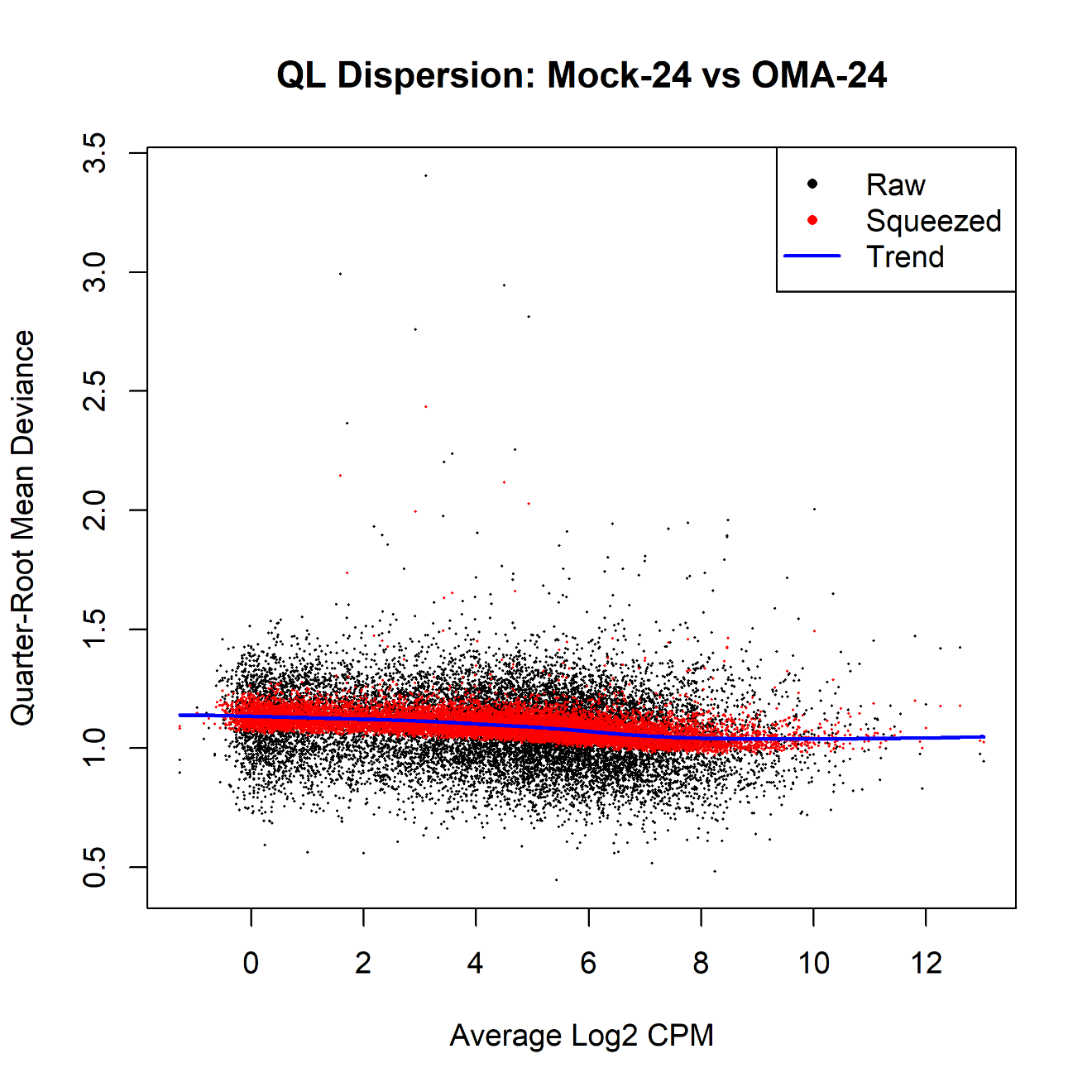


**Figure S2. Quasi-likelihood dispersion plot for the Mock-24 vs OMA-24 contrast.** Raw (black) and squeezed (red) quarter-root mean deviances are plotted against average log₂ CPM, with the fitted mean–variance trend shown in blue. The dispersion structure shows consistent shrinkage toward the trend and no abnormal overdispersion, which confirms that the data are appropriate for GLM quasi-likelihood differential expression testing.


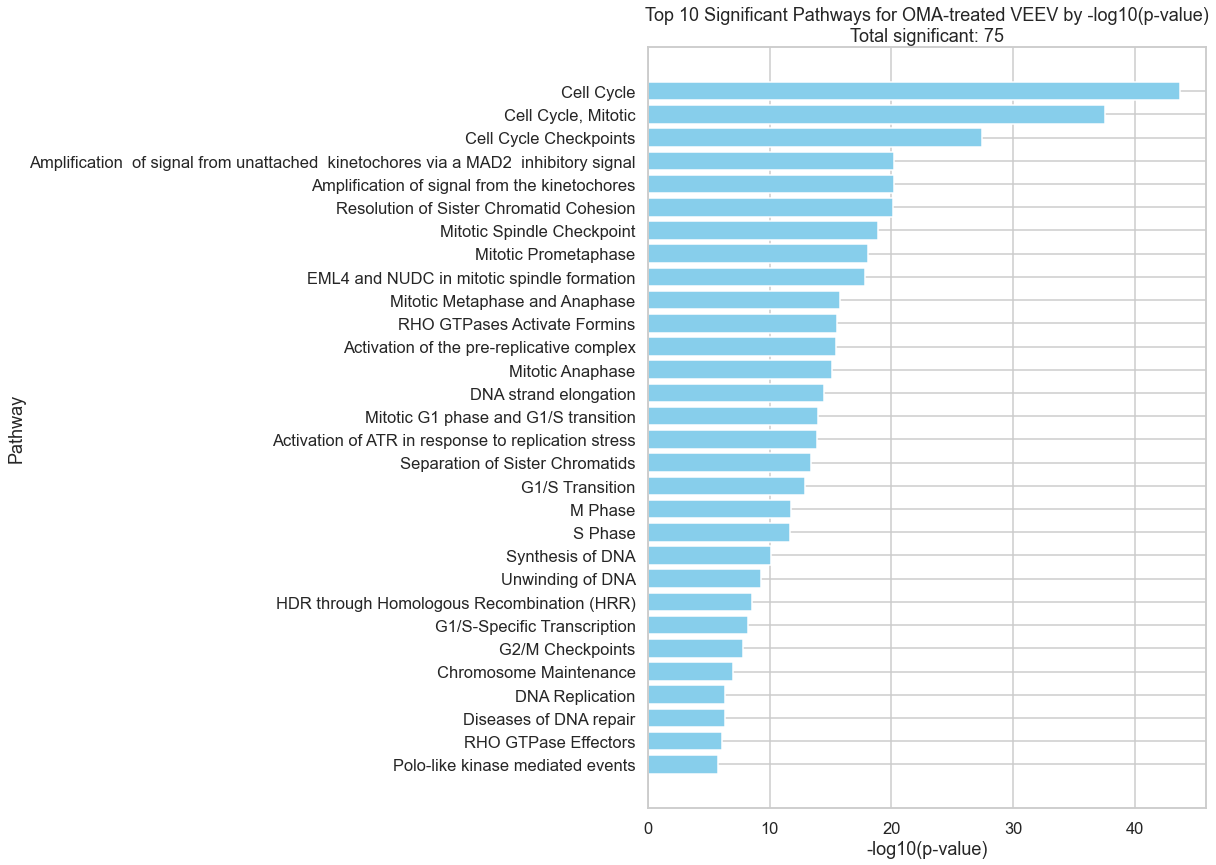


***Figure S3. Reactome pathway enrichment analysis of significantly downregulated genes in OMA treated VEEV infected HUVECs at 24 hpi.*** *Bar plot showing the top enriched Reactome pathways derived from genes significantly downregulated under OMA treatment compared to mock (BH adjusted p < 0.05 and log₂FC < -1). Pathways predominantly relate to cell cycle regulation, mitotic progression, DNA replication, and checkpoint control, which indicates broad suppression of proliferative programs rather than targeted modulation of antiviral or immune responses. Bars represent −log₁₀(p-value), and a total of 75 pathways met significance criteria.*
